# Cognitive Function in Parkinson's Disease Patients with and without Anxiety

**DOI:** 10.1155/2016/6254092

**Published:** 2016-10-09

**Authors:** K. A. Ehgoetz Martens, J. Y. Y. Szeto, A. J. Muller, J. M. Hall, M. Gilat, C. C. Walton, S. J. G. Lewis

**Affiliations:** Brain and Mind Centre, Faculty of Medicine, University of Sydney, 100 Mallet Street, Camperdown, Sydney, NSW 2050, Australia

## Abstract

Research on the implications of anxiety in Parkinson's disease (PD) has been neglected despite its prevalence in nearly 50% of patients and its negative impact on quality of life. Previous reports have noted that neuropsychiatric symptoms impair cognitive performance in PD patients; however, to date, no study has directly compared PD patients with and without anxiety to examine the impact of anxiety on cognitive impairments in PD. This study compared cognitive performance across 50 PD participants with and without anxiety (17 PDA+; 33 PDA−), who underwent neurological and neuropsychological assessment. Group performance was compared across the following cognitive domains: simple attention/visuomotor processing speed, executive function (e.g., set-shifting), working memory, language, and memory/new verbal learning. Results showed that PDA+ performed significantly worse on the Digit Span forward and backward test and Part B of the Trail Making Task (TMT-B) compared to the PDA− group. There were no group differences in verbal fluency, logical memory, or TMT-A performance. In conclusion, anxiety in PD has a measurable impact on working memory and attentional set-shifting.

## 1. Introduction

Anxiety affects quality of life in those living with Parkinson's disease (PD) more so than overall cognitive status, motor deficits, apathy, and depression [1–3]. Although anxiety and depression are often related and coexist in PD patients [4], recent research suggests that anxiety rather than depression is the most prominent and prevalent mood disorder in PD [5, 6]. Yet, our current understanding of anxiety and its impact on cognition in PD, as well as its neural basis and best treatment practices, remains meager and lags far behind that of depression [7].

Overall, neuropsychiatric symptoms in PD have been shown to be negatively associated with cognitive performance. For example, higher depression scores have been correlated with lower scores on the Mini-Mental State Exam (MMSE) [8, 9] as well as tests of memory and executive functions (e.g., attention) [10–14]. Likewise, apathy and anhedonia in PD patients have been associated with executive dysfunction [10, 15–23]. However, few studies have specifically investigated the relationship between anxiety and cognition in PD.

One study showed a strong negative relationship between anxiety (both state and trait) and overall cognitive performance (measured by the total of the repeatable battery for the assessment of neuropsychological status index) within a sample of 27 PD patients [24]. Furthermore, trait anxiety was negatively associated with each of the cognitive domains assessed by the RBANS (i.e., immediate memory, visuospatial construction, language, attention, and delayed memory). Two further studies have examined whether anxiety differentially affects cognition in patients with left-sided dominant PD (LPD) versus right-sided dominant PD (RPD); however, their findings were inconsistent. The first study found that working memory performance was worse in LPD patients with anxiety compared to RPD patients with anxiety [25], whereas the second study reported that, in LPD, apathy but not anxiety was associated with performance on nonverbally mediated executive functions and visuospatial tasks (e.g., TMT-B, WMS-III spatial span), while in RPD, anxiety but not apathy significantly correlated with performance on verbally mediated tasks (e.g., clock reading test and Boston naming test) [15]. Furthermore, anxiety was significantly correlated with neuropsychological measures of attention and executive and visuospatial functions [15]. Taken together, it is evident that there are limited and inconsistent findings describing the relationship between anxiety and cognition in PD and more specifically how anxiety might influence particular domains of cognition such as attention and memory and executive functioning. It is also striking that, to date, no study has examined the influence of anxiety on cognition in PD by directly comparing groups of PD patients with and without anxiety while excluding depression. This was the primary objective of the current study.

Given that research on healthy young adults suggests that anxiety reduces processing capacity and impairs processing efficiency, especially in the central executive and attentional systems of working memory [26, 27], we hypothesized that PD patients with anxiety would show impairments in attentional set-shifting and working memory compared to PD patients without anxiety. Furthermore, since previous work, albeit limited, has focused on the influence of symptom laterality on anxiety and cognition, we also explored this relationship.

## 2. Methods

Seventeen PD patients with anxiety and thirty-three PD patients without anxiety were included in this study (see Table 1). The cross-sectional data from these participants was taken from a patient database that has been compiled over the past 8 years (since 2008) at the Parkinson's Disease Research Clinic at the Brain and Mind Centre, University of Sydney. Inclusion criteria involved a diagnosis of idiopathic PD according to the United Kingdom Parkinson's Disease Society Brain Bank criteria [28] and were confirmed by a neurologist (SJGL). Patients also had to have an adequate proficiency in English and have completed a full neuropsychological assessment. Ten patients in this study (5 PD with anxiety; 5 PD without anxiety) were taking psychotropic drugs (i.e., benzodiazepine or selective serotonin reuptake inhibitor). Patients with an MMSE of less than 24 were excluded. Patients were also excluded if they had other neurological disorders, psychiatric disorders other than affective disorders (such as anxiety), or if they reported a score greater than six on the depression subscale of the Hospital Anxiety and Depression Scale (HADS). Thus, all participants who scored within a “depressed” (HADS-D > 6) range were excluded from this study, in attempt to examine a refined sample of PD patients with and without anxiety in order to determine the independent effect of anxiety on cognition. This research was approved by the Human Research Ethics Committee of the University of Sydney, and written informed consent was obtained from all participants.

Self-reported HADS was used to assess anxiety in PD and has been previously shown to be a useful measure of clinical anxiety in PD [29]. A cut-off score of >8 on the anxiety subscale of the HADS (HADS-A) was used to identify PD cases with anxiety (PDA+), while a cut-off score of <6 on the HADS-A was used to identify PD cases without anxiety (PDA−). This criterion was more stringent than usual (>7 cut-off score) [30], in effort to create distinct patient groups.

All participants underwent a neurological assessment conducted while on their medications. The neurological evaluation rated participants according to Hoehn and Yahr (H&Y) stages [31] and assessed their motor symptoms using part III of the revised MDS Task Force Unified Parkinson's Disease Rating Scale (UPDRS) [32]. In a similar way, symptom laterality was determined by taking a left/right ratio. This was determined by calculating a total left and right score from rigidity items 30–35, voluntary movement items 36–43, and tremor items 50–57 from the MDS-UPDRS part III (see Table 1).

Neuropsychological functioning was assessed using standardized tests and appropriate normative data. A detailed description of the neuropsychological battery was reported elsewhere [33]. Processing speed was assessed using the Trail Making Test, Part A (TMT-A,* z*-score) [34]. Attentional set-shifting was measured using the Trail Making Test, Part B (TMT-B,* z*-score) [34]. Working memory was assessed using the Digit Span forward and backward subtest of the Wechsler Memory Scale-III (raw scores) [35]. Language was assessed with semantic and phonemic verbal fluency via the Controlled Oral Word Associated test (COWAT animals and letters,* z*-score) [36]. The ability to retain learned verbal memory was assessed using the Logical Memory subtest from the Wechsler Memory Scale-III (LM-I* z*-score, LM-II* z*-score, % LM retention* z*-score) [35]. The Mini-Mental State Examination (MMSE) was used to assess global cognition.

### 2.1. Statistical Analyses

The Shapiro-Wilk test was performed to determine normal distribution. Demographic, clinical, and neuropsychological variables were compared between the two groups with the independent* t*-test or Mann–Whitney *U* test, depending on whether the variable met parametric assumptions. Chi-square tests were used to examine gender and symptom laterality differences between groups. All analyses employed an alpha level of *p* < 0.05 and were two-tailed. Spearman correlations were performed separately in each group to examine associations between anxiety and/or depression ratings and cognitive functions.

## 3. Results

### 3.1. Demographic and Clinical Characteristics

As expected, the PDA+ group reported significant greater levels of anxiety on the HADS-A (*U* = 0, *p* < 0.001) and higher total score on the HADS (*U* = 1, *p* < 0.001) compared to the PDA− group (Table 1). Groups were matched in age (*t*(48) = 1.31, *p* = 0.20), disease duration (*U* = 259, *p* = 0.66), UPDRS-III score (*U* = 250.5, *p* = 0.65), H&Y (*U* = 245, *p* = 0.43), LEDD (*U* = 159.5, *p* = 0.80), and depression (HADS-D) (*U* = 190.5, *p* = 0.06). Additionally, all groups were matched in the distribution of gender (*χ* ^2^ = 0.098, *p* = 0.75) and side-affected (*χ* ^2^ = 0.765, *p* = 0.38).

### 3.2. Cognitive Functioning

**Figure 1:**
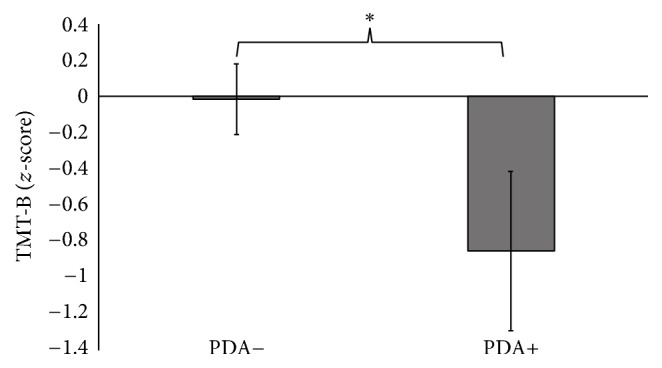
PD patients with anxiety performed significantly worse on Part B of the Trail Making Task compared to PD patients without anxiety. *∗* denotes *p* values < 0.05. Error bars represent standard error.

**Figure 2:**
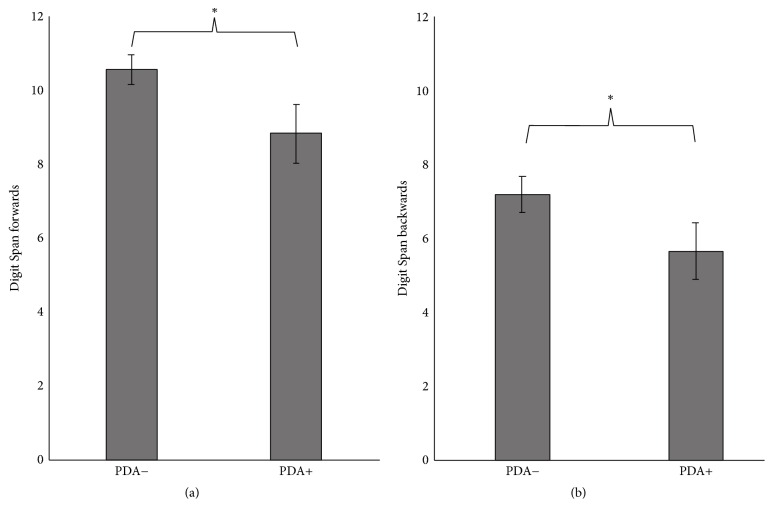
PD patients with anxiety performed significantly worse on the Digit Span forward (a) and backward (b) test compared to PD patients without anxiety. *∗* denotes *p* values < 0.05. Error bars represent standard error of the means.

There were no group differences for TMT-A performance (*U* = 256, *p* = 0.62) (Table 2); however, the PDA+ group had worse performance on the Trail Making Test Part B (*t*(46) = 2.03, *p* = 0.048) compared to the PDA− group (Figure 1). The PDA+ group also demonstrated significantly worse performance on the Digit Span forward subtest (*t*(48) = 2.22, *p* = 0.031) and backward subtest (*U* = 190.5, *p* = 0.016) compared to the PDA− group (Figures 2(a) and 2(b)). Neither semantic verbal fluency (*t*(47) = 0.70, *p* = 0.49) nor phonemic verbal fluency (*t*(47) = 0.39, *p* = 0.70) differed between groups. Logical Memory I immediate recall test (*U* = 176, *p* = 0.059) showed a trend that the PDA+ group had worse new verbal learning and immediate recall abilities than the PDA− group. However, Logical Memory II test performance (*U* = 219, *p* = 0.204) and Logical Memory % retention (*U* = 242.5, *p* = 0.434) did not differ between groups. There were also no differences between groups in global cognition (MMSE) (*U* = 222.5, *p* = 0.23).

### 3.3. Comparison within LPD and RPD Patients

Participants were split into LPD and RPD, and then further group differences were examined between PDA+ and PDA−. Importantly, the groups remained matched in age, disease duration, UPDRS-III, DDE, H&Y stage, and depression but remained significantly different on self-reported anxiety. LPDA+ demonstrated worse performance on the Digit Span forward test (*t*(19) = 2.29, *p* = 0.033) compared to LPDA−, whereas RPDA+ demonstrated worse performance on the Digit Span backward test (*U* = 36.5, *p* = 0.006), LM-I immediate recall (*U* = 37.5, *p* = 0.008), and LM-II (*U* = 45.0, *p* = 0.021) but not LM % retention (*U* = 75.5, *p* = 0.39) compared to RPDA−.

## 4. Discussion

This study is the first to directly compare cognition between PD patients with and without anxiety. The findings confirmed our hypothesis that anxiety negatively influences attentional set-shifting and working memory in PD. More specifically, we found that PD patients with anxiety were more impaired on the Trail Making Test Part B which assessed attentional set-shifting, on both Digit Span Tests which assessed working memory and attention, and to a lesser extent on the Logical Memory test which assessed memory and new verbal learning compared to PD patients without anxiety. Taken together, these findings suggest that anxiety in PD may reduce processing capacity and impair processing efficiency, especially in the central executive and attentional systems of working memory in a similar way as seen in young healthy adults [26, 27].

Although the neurobiology of anxiety in PD remains unknown, many researchers have postulated that anxiety disorders are related to neurochemical changes that occur during the early, premotor stages of PD-related degeneration [37, 38] such as nigrostriatal dopamine depletion, as well as cell loss within serotonergic and noradrenergic brainstem nuclei (i.e., raphe nuclei and locus coeruleus, resp., which provide massive inputs to corticolimbic regions). Over time, chronic dysregulation of adrenocortical and catecholamine functions can lead to hippocampal damage as well as dysfunctional prefrontal neural circuitries [39, 40], which play a key role in memory and attention [24]. Recent functional neuroimaging work has suggested that enhanced hippocampal activation during executive functioning and working memory tasks may represent compensatory processes for impaired frontostriatal functions in PD patients compared to controls [41]. Therefore, chronic stress from anxiety, for example, may disrupt compensatory processes in PD patients and explain the cognitive impairments specifically in working memory and attention seen in PD patients with anxiety. It has also been suggested that hyperactivation within the putamen may reflect a compensatory striatal mechanism to maintain normal working memory performance in PD patients; however, losing this compensatory activation has been shown to contribute to poor working memory performance [42]. Anxiety in mild PD has been linked to reduced putamen dopamine uptake which becomes more extensive as the disease progresses [43]. This further supports the notion that anxiety may disrupt compensatory striatal mechanisms as well, providing another possible explanation for the cognitive impairments observed in PD patients with anxiety in this study.

Noradrenergic and serotonergic systems should also be considered when trying to explain the mechanisms by which anxiety may influence cognition in PD. Although these neurotransmitter systems are relatively understudied in PD cognition, treating the noradrenergic and serotonergic systems has shown beneficial effects on cognition in PD. Selective serotonin reuptake inhibitor, Citalopram, was shown to improve response inhibition deficits in PD [44], while noradrenaline reuptake blocker, atomoxetine, has been recently reported to have promising effects on cognition in PD [45, 46].

Overall, very few neuroimaging studies have been conducted in PD in order to understand the neural correlates of PD anxiety and its underlying neural pathology. Future research should focus on relating anatomical changes and neurochemical changes to neural activation in order to gain a clearer understanding on how these pathologies affect anxiety in PD. To further understand how anxiety and cognitive dysfunction are related, future research should focus on using advanced structural and function imaging techniques to explain both cognitive and neural breakdowns that are associated with anxiety in PD patients. Research has indicated that those with amnestic mild cognitive impairment who have more neuropsychiatric symptoms have a greater risk of developing dementia compared to those with fewer neuropsychiatric symptoms [10]. Future studies should also examine whether treating neuropsychiatric symptoms might impact the progression of cognitive decline and improve cognitive impairments in PD patients.

### 4.1. Does Symptom Laterality Influence the Effect of Anxiety on Cognition?

Previous studies have used PD symptom laterality as a window to infer asymmetrical dysfunction of neural circuits. For example, LPD patients have greater inferred right hemisphere pathology, whereas RPD patients have greater inferred left hemisphere pathology [15]. Thus, cognitive domains predominantly subserved by the left hemisphere (e.g., verbally mediated tasks of executive function and verbal memory) might be hypothesized to be more affected in RPD than LPD; however, this remains controversial [47]. It has also been suggested that since anxiety is a common feature of left hemisphere involvement [48, 49], cognitive domains subserved by the left hemisphere may also be more strongly related to anxiety [15]. Results from this study showed selective verbal memory deficits in RPD patients with anxiety compared to RPD without anxiety, whereas LPD patients with anxiety had greater attentional/working memory deficits compared to LPD without anxiety. Although these results align with previous research [15], interpretations of these findings should be made with caution due to the small sample size in the LPD comparison specifically.

### 4.2. Limitations, Considerations, and Future Directions

We would like to acknowledge a few shortcomings of this study. Recent work has suggested that the HADS questionnaire may underestimate the burden of anxiety related symptomology and therefore be a less sensitive measure of anxiety in PD [30, 50]. In addition, our small sample size also limited the statistical power for detecting significant findings. Based on these limitations, our findings are likely conservative and underrepresent the true impact anxiety has on cognition in PD. Additionally, the current study employed a very brief neuropsychological assessment including one or two tests for each cognitive domain. Future studies are encouraged to collect a more complex and comprehensive battery from a larger sample of PD participants in order to better understand the role anxiety plays on cognition in PD.

Another limitation of this study was the absence of diagnostic interviews to characterize participants' psychiatric symptoms and specify the type of anxiety disorders included in this study. Future studies should perform diagnostic interviews with participants (e.g., using DSM-V criteria) rather than relying on self-reported measures to group participants, in order to better understand whether the type of anxiety disorder (e.g., social anxiety, phobias, panic disorders, and generalized anxiety) influences cognitive performance differently in PD.

One advantage the HADS questionnaire provided over other anxiety scales was that it assessed both anxiety and depression simultaneously and allowed us to control for coexisting depression. Although there was a trend that the PDA+ group self-reported higher levels of depression than the PDA− group, all participants included in the study scored <6 on the depression subscale of the HADS. Controlling for depression while assessing anxiety has been identified as a key shortcoming in the majority of recent work [10]. Considering many previous studies have investigated the influence of depression on cognition in PD without accounting for the presence of anxiety and the inconsistent findings reported to date, we recommend that future research should try to disentangle the influence of anxiety versus depression on cognitive impairments in PD.

Considering the growing number of clinical trials for treating depression, there are few if any for the treatment of anxiety in PD. Anxiety is a key contributor to decreased quality of life in PD and greatly requires better treatment options. Moreover, anxiety has been suggested to play a key role in freezing of gait (FOG) [51], which is also related to attentional set-shifting [52, 53]. Future research should examine the link between anxiety, set-shifting, and FOG, in order to determine whether treating anxiety might be a potential therapy for improving FOG.

## Figures and Tables

**Table 1:** Demographic and clinical variables in PD participants.

Variable	PDA− (*N* = 33)	PDA+ (*N* = 17)	*p* values
*Demographic*			
Age (years)	68.3 (8.9)	64.9 (8.1)	*p* = 0.20
Gender (% male)	19/14 (57.6%)	9/8 (52.9%)	*p* = 0.75
*Clinical*			
Disease duration (years)	6.6 (4.2)	6.6 (5.6)	*p* = 0.66
UPDRS-III	31.9 (13.0)	31.2 (15.3)	*p* = 0.65
LPD/RPD ratio (% LPD)	15/17 (45.5%)	6/11 (35.3%)	*p* = 0.38
H&Y stage, median (range)	2.0 (1–4)	2.0 (1–5)	*p* = 0.43
LEDD, mg/day	674.0 (550.5)	710.2 (478.7)	*p* = 0.80
HADS-A	2.03 (1.6)	10.5 (1.9)	^*∗*^ *p* < 0.001
HADS-D	3.2 (1.7)	4.1 (1.6)	*p* = 0.06
HADS-total	5.2 (2.7)	14.7 (2.9)	^*∗*^ *p* < 0.001

Note: values reflect mean (SD). LEDD: levodopa equivalent daily dose. *∗* indicates significant group differences.

**Table 2:** Neuropsychological performance.

	PDA− (*N* = 33)	PDA+ (*N* = 17)	*p* values
*Global cognition*			
MMSE	28.2 (1.5)	27.5 (1.8)	*p* = 0.23
*Attention/visuomotor processing speed*			
TMT-A, *z*-score	−0.15 (1.2)	0.66 (2.23)	*p* = 0.62
*Executive function*			
TMT-B, *z*-score	−0.02 (1.1)	−0.85 (1.8)	^*∗*^ *p* = 0.048
*Working memory*			
Digit Span forward total	10.6 (2.3)	8.8 (3.2)	^*∗*^ *p* = 0.031
Digit Span backward total	7.2 (2.8)	5.7 (1.5)	^*∗*^ *p* = 0.012
*Language*			
Semantic verbal fluency, *z*-score	−0.04 (1.0)	−0.26 (1.1)	*p* = 0.49
Phonemic verbal fluency, *z*-score	−0.10 (1.1)	−0.23 (1.2)	*p* = 0.70
*Memory/new verbal learning*			
LM-I immediate recall, *z*-score	−0.21 (1.1)	−0.83 (1.1)	*p* = 0.059
LM-II, *z*-score	0.10 (1.1)	−0.29 (1.1)	*p* = 0.20
% LM retention, *z*-score	0.40 (1.2)	0.14 (1.2)	*p* = 0.43

Note: values represent mean (SD). *∗* indicates significant differences at the *p* < 0.05 level.
